# Supplementary material for: Internalisation and Biological Activity of Nucleic Acids Delivering Cell-Penetrating Peptide Nanoparticles Is Controlled by the Biomolecular Corona
Source: Pharmaceuticals (Basel). 2021 Jul 12;14(7):667. doi: 10.3390/ph14070667 (PMC8308718; doi:10.3390/ph14070667)
Supplement: Supplementary file 1 [file pharmaceuticals-14-00667-s001.zip › SupplFig1_6.pdf]

Supplementary figures to:

“**Internalisation and biological activity of nucleic acids delivering cell-penetrating peptide nanoparticles is controlled by the biomolecular corona**”

by Annely Lorents, Maria Maloverjan, Kärt Padari, Margus Pooga

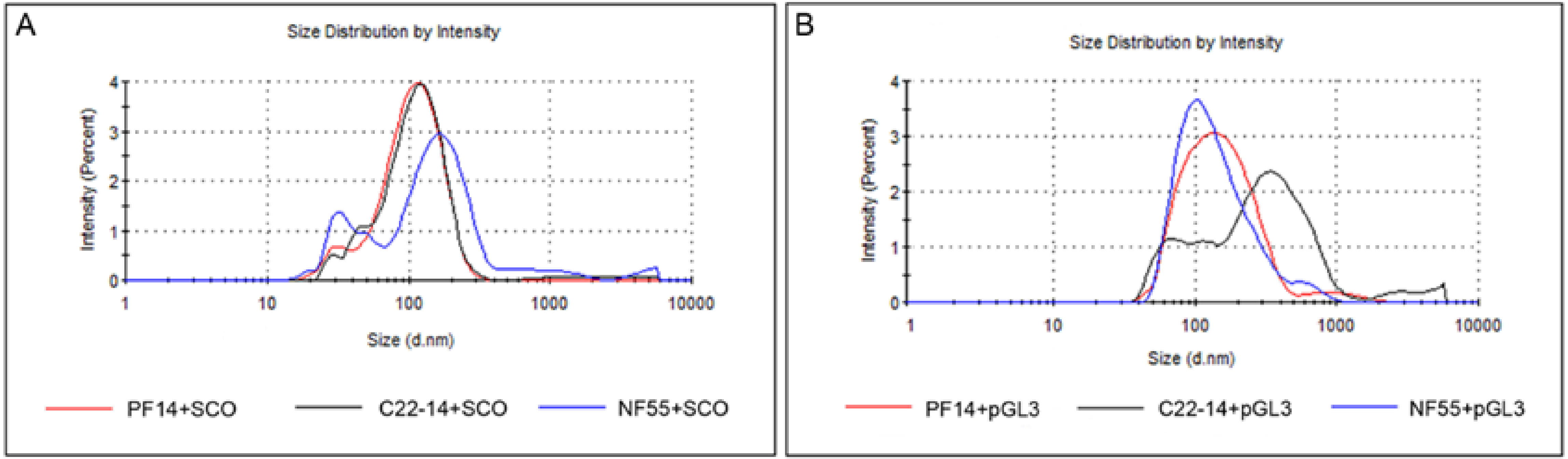

Supplementary Figure 1. Size of CPP-nucleic acid nanoparticles analysed by Dynamic Light Scattering. Size distribution of CPP-SCO (A) and CPP-pDNA (B) nanoparticles represent the mean of four DLS measurements. CPP-SCO nanoparticles were formed at molar ratio 5:1 and measured at 0.5 µM SCO concentration using Zetasizer Nano ZS (Malvern Instruments). CPP-pDNA nanoparticles were formed at N/P ratio 2 in water and measured at 0.2 µg/ml pDNA concentration.

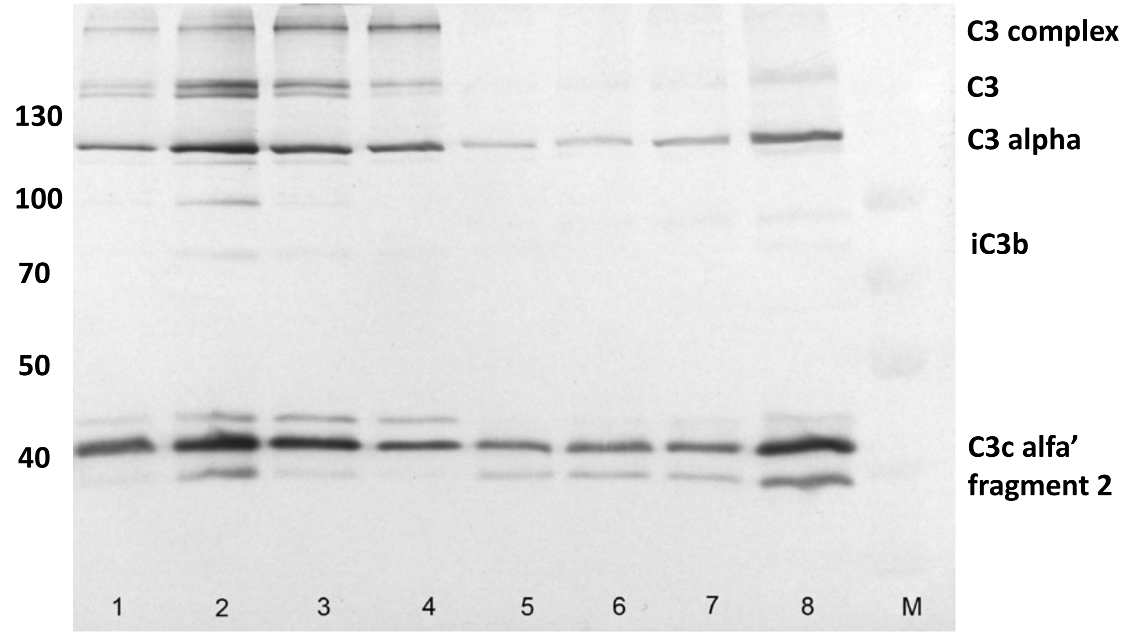

Supplementary Figure 1. Analysis of complement component 3 (C3) and its fragments’ level in protein corona of plasmid DNA and CPP-pDNA nanoparticles. CPP-pGL3 nanoparticles or pGL3 alone were immobilized on streptavidin-superparamagnetic beads, or beads were left untreated (control) before incubation with mouse plasma or serum. After pull-down, bound C3 subunits and its fragments were separated by electrophoresis on a 9% SDS PAGE, transferred to PVDF) membrane and visualised by Western blot using rabbit monoclonal anti-C3 antibody. One representative of at least three independent experiments is presented.

Lines:

1) Empty streptavidin-beads in mouse plasma, 60 min incubation
2) b-pDNA NPs in mouse plasma, 60 min incubation
3) PF14-b-pDNA NPs in mouse plasma, 60 min incubation
4) PF14-b-pDNA NPs in mouse plasma, 5 min incubation
5) Empty streptavidin-beads in mouse serum, 60 min incubation
6) PF14-b-pDNA NPs in mouse serum, 5 min incubation
7) PF14-b-pDNA NPs in mouse serum, 60 min incubation
8) b-pDNA NPs in mouse serum, 60 min incubation

M) Marker

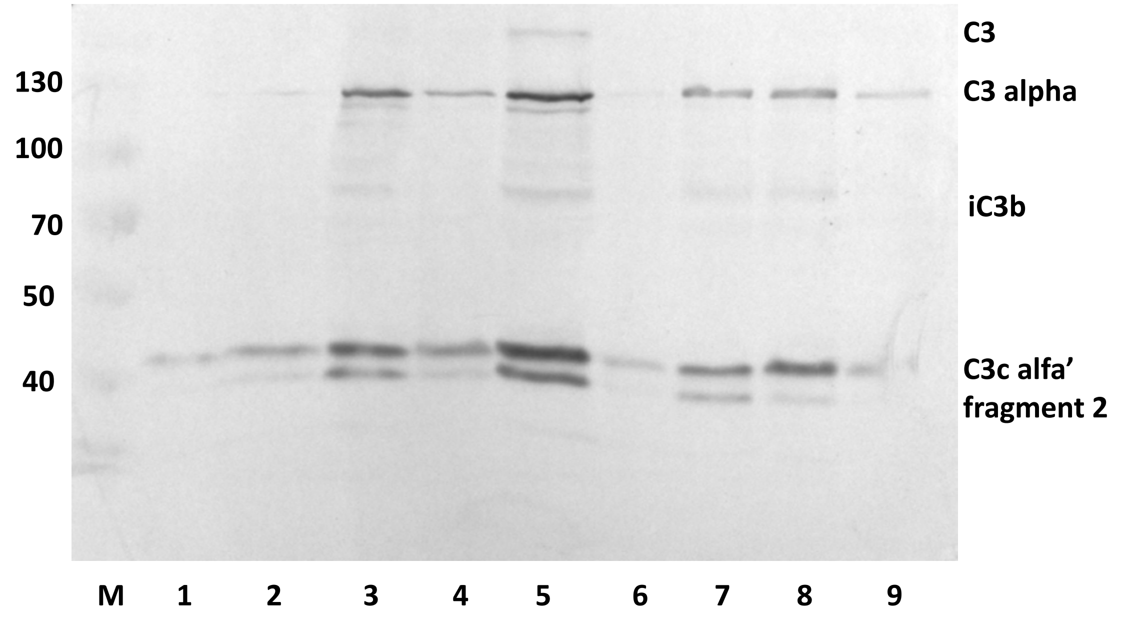

Supplementary Figure 2. Analysis of complement component 3 (C3) and its fragments' level in protein corona of plasmid DNA and CPP-pDNA nanoparticles. CPP-pGL3 nanoparticles or pGL3 alone were immobilized on streptavidin-superparamagnetic beads, or beads were left untreated (control) before incubation with mouse plasma. After pull-down, bound C3 subunits and its fragments were separated by electrophoresis on a 9% SDS PAGE, transferred to PVDF) membrane and visualised by Western blot using rabbit monoclonal anti-C3 antibody. One representative of at least three independent experiments is presented.

Lines:
M) Marker
1) PF14-b-pDNA NPs in mouse plasma, 5 min incubation
2) PF14-b-pDNA NPs in mouse plasma, 60 min incubation
3) NF55-b-pDNA NPs in mouse plasma, 60 min incubation
4) C22-PF14-b-pDNA NPs in mouse plasma, 60 min incubation
5) b-pDNA NPs in mouse plasma, 60 min incubation
6) Empty streptavidin-beads in mouse plasma, 60 min incubation
7) Mouse plasma
8) Mouse serum
9) Empty streptavidin-beads in mouse serum, 60 min incubation

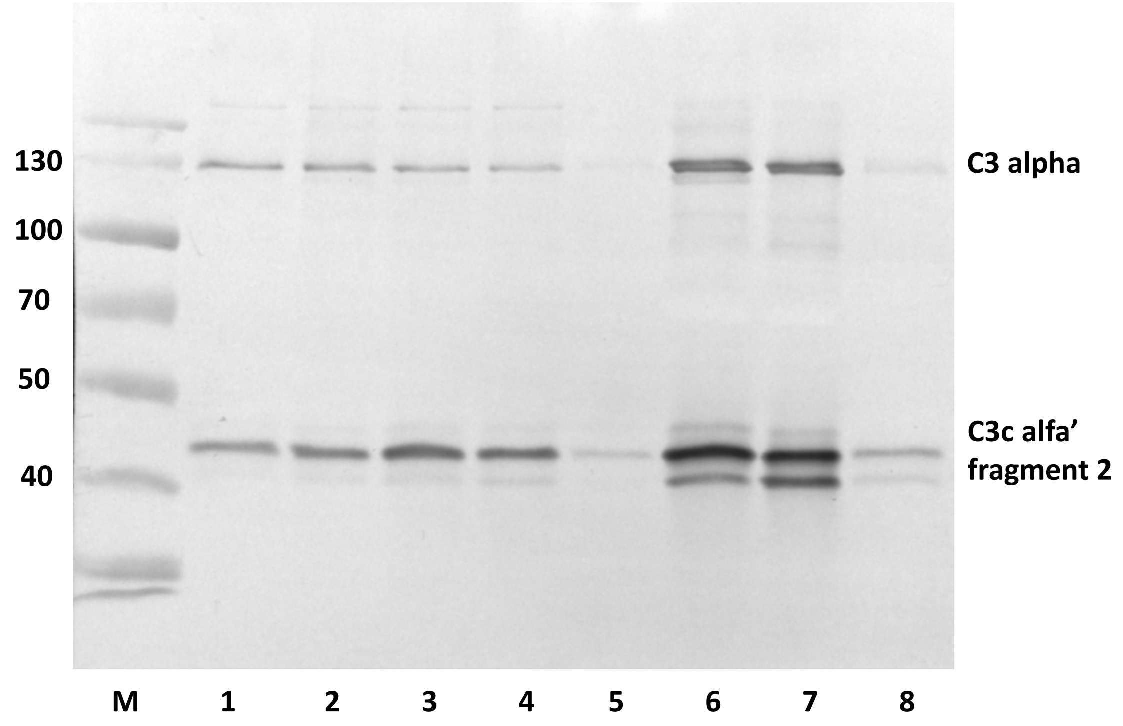

Supplementary Figure 3. Analysis of complement component 3 (C3) and its fragments’ level in protein corona of SCO DNA and CPP-SCO nanoparticles. CPP-SCO nanoparticles or SCO alone were immobilized on streptavidin-superparamagnetic beads, or beads were left untreated (control) before incubation with mouse plasma. After pull-down, bound C3 subunits and its fragments were separated by electrophoresis on a 9% SDS PAGE, transferred to PVDF) membrane and visualised by Western blot using rabbit monoclonal anti-C3 antibody. One representative of at least three independent experiments is presented.

Lines:

M) Marker
1) PF14- b-SCO NPs in mouse serum, 5 min incubation
2) PF14-b-SCO NPs in mouse serum, 60 min incubation
3) NF55-b-SCO NPs in mouse serum, 60 min incubation
4) b-SCO NPs in mouse serum, 60 min incubation
5) Empty streptavidin-beads in mouse serum, 60 min incubation
6) Mouse serum
7) Mouse plasma
8) Empty streptavidin-beads in mouse plasma, 60 min incubation

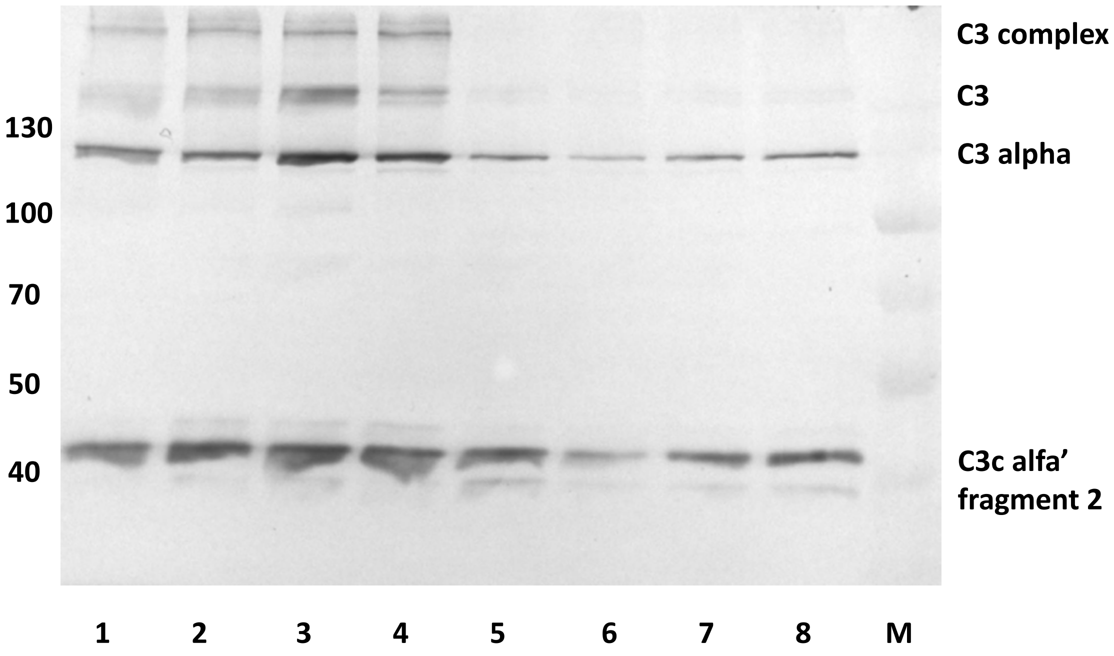

Supplementary Figure 4. Analysis of complement component 3 (C3) and its fragments’ level in protein corona of SCO DNA and CPP-SCO nanoparticles. CPP-SCO nanoparticles or SCO alone were immobilized on streptavidin-superparamagnetic beads, or beads were left untreated (control) before incubation with mouse plasma or serum . After pull-down, bound C3 subunits and its fragments were separated by electrophoresis on a 9% SDS PAGE, transferred to PVDF) membrane and visualised by Western blot using rabbit monoclonal anti-C3 antibody. One representative of at least three independent experiments is presented.

Lines:
1) Empty streptavidin-beads in mouse plasma, 60 min incubation
2) b-SCO NPs in mouse plasma, 60 min incubation
3) PF14-b-SCO NPs in mouse plasma, 60 min incubation
4) PF14- b-SCO NPs in mouse plasma, 5 min incubation
5) Empty streptavidin-beads in mouse serum, 60 min incubation
6) b-SCO NPs in mouse serum, 60 min incubation
7) PF14- b-SCO NPs in mouse serum, 5 min incubation
8) PF14- b-SCO NPs in mouse serum, 60 min incubation
M) Marker

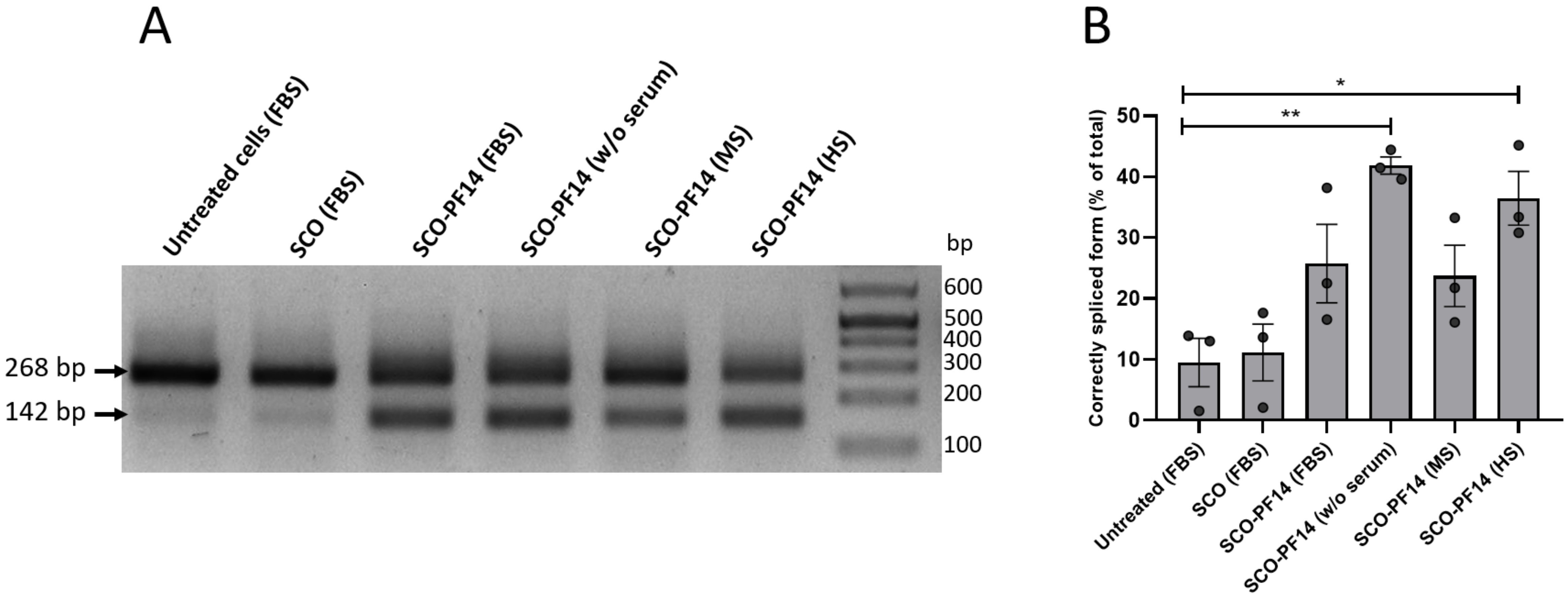

Supplementary Figure S6. Correction of luciferase mRNA splicing by SCO delivered into cells in nanoparticles with PF14 carrying different protein corona. HeLa pLuc705 cells were incubated for 4 h with nanoparticles (100 nM SCO, 500 nM PF14) in media containing different sera (5% v:v) or not containing serum. Cells were harvested after additional 20 h of incubation in standard medium (with 10% FBS). The switching of the luciferase mRNA splicing by SCO was analysed by RT PCR and the resulting products were visualised in agarose gel and quantified using ImageJ software. Each dataset in panel B represents mean +/- SEM of 3 experiments. Data was analysed by one-way ANOVA with Tukey's test. Asterisks indicate statistically significant difference between two datasets, *p-value < 0.05, **p-value < 0.005. FBS, foetal bovine serum; MS, murine serum; HS, human serum.

PCR analysis of splicing correction efficiency in the presence of different sera

HeLa pLuc705 cells were seeded on a 12-well plate (80 000 cells per well). Next day, SCO or SCO-PF14 nanoparticles (1 µM SCO, 5 µM PF14) were prepared in Milli-Q water. Nanoparticles were diluted 10-fold in DMEM containing sera of different animals (5% v:v) or not containing serum and added to the cells for 4 hours. Cells were harvested after additional 20 h of incubation in standard medium (DMEM supplemented with 10% FBS). Total RNA was extracted using the RNeasy Mini Kit (Qiagen). RNA was subjected to DNase treatment (TURBO DNase, Thermo Scientific) and cDNA was synthesized from 1.34 µg of RNA using RevertAid First Strand cDNA Synthesis Kit (Thermo Scientific). PCR of luciferase (with primers Ex49F 5′-AAACCAAGCACTCAGCCAGT-3′ and Ex54R 5′-CAGCAGAATAGTCCCGAAGAA-3′, Kuhn, J. et al. 2019) mRNA splicing and GAPDH gene (used as a housekeeper) was performed using Platinum II Hot-Start Green PCR Master Mix (Invitrogen). Per 20 µl reaction, 1.2 µl of cDNA was used.

PCR program:

94 ^{o}C	2 min	1x
94 ^{o}C	15 s	
60 ^{o}C	15 s	40x
68 ^{o}C	40 s	

Agarose gel electrophoresis was performed and resulting bands were quantified using ImageJ software. For normalization, the GAPDH gene was used.
